# The Influence of Judgments of Learning on Collaborative Memory for Items and Sequences

**DOI:** 10.3390/bs15070905

**Published:** 2025-07-03

**Authors:** Xiaochun Luo, Qian Xiao, Weihai Tang

**Affiliations:** 1 Department of Student Affairs, Sichuan College of Architectural Technology, Chengdu 610399, China; luoxiaochun@scac.edu.cn; 2 Faculty of Psychology, Tianjin Normal University, Tianjin 300387, China; 2201340028@stu.tjnu.edu.cn; 3 College of Education Science, Henan Institute of Science and Technology, Xinxiang 453003, China; 4 School of Sociology, University of Sanya, Sanya 572022, China

**Keywords:** judgments of learning (JOLs), sequential reconstruction, collaborative memory, reactivity effect, collaborative inhibition

## Abstract

The present study examined how making judgments of learning (JOLs) vs. not making judgments of learning (no-JOLs) influences item and sequential memory in collaborative contexts. According to the item-order hypothesis, making JOLs improves memory for specific items (i.e., item memory) but disrupts sequential memory where memory for temporal relationships between items is required. If JOLs do enhance item memory performance, the study predicts they may effectively eliminate collaborative inhibition through a compensatory enhancement mechanism. Specifically, the magnitude of JOL-induced memory improvement appears to be greater in collaborative groups than in nominal groups. This differential enhancement likely offsets the typical memory impairment caused by collaborative retrieval interference, resulting in statistically equivalent final performance between groups. Consequently, the collaborative inhibition effect may disappear under JOL conditions. This study employed a 2 (group: collaborative vs. nominal; between-subjects) × 2 (metamemory monitoring: with vs. without judgments of learning; within-subjects) × 2 (test type: recognition vs. sequential reconstruction; within-subjects) mixed factorial design. The findings indicated that making judgments of learning significantly enhanced item memory performance while having no noticeable effect on sequential memory. It suggests that the reactivity effect is only present in item memory. Additionally, it was found that both item recognition and sequential memory performance were lower in the collaborative group compared with the nominal group, highlighting the presence of collaborative inhibition. These results suggest that the reactivity effect and collaborative inhibition are two distinct memory phenomena that do not affect each other.

## 1. Introduction

A judgment of learning (JOL) is one’s judgment about how well a given item is learned and how likely this item will be successfully retrieved on a future memory test (Nelson, 1990). Making judgments of learning during the learning process helps monitor the extent of learning and influences recall performance, a phenomenon known as the reactivity effect (Dougherty et al., 2018; Mitchum et al., 2016). The findings from current research on the reactivity effects of JOL are inconsistent; some studies have reported positive reactivity effects (Rivers et al., 2023; Spellman & Bjork, 1992). However, other studies found negative reactivity effects (Mitchum, 2011; Mitchum et al., 2016), while some did not find any reactivity effects (Mitchum et al., 2016; Zhao et al., 2023). Furthermore, the results of various meta-analyses have been inconsistent (Double et al., 2018; Xu et al., 2022). The primary reasons for the inconsistent results are the variations in experimental materials used (such as educational texts, relevant word pairs, irrelevant words, and mixed word pairs), differences in learning duration (including other-controlled pacing and self-controlled pacing), the timing of judgments of learning (JOL), which can be immediate or delayed, and variations in JOL durations (ranging from 500 ms to 4 or 5 s).

Remembering the sequence of events is ubiquitous in everyday life, and people memorize the order of words uttered in a conversation, the sequence of numbers in a telephone number, and the series of turns made when driving to a destination. Some psychologists believe that all memory is essentially memorizing sequences (Thomas et al., 2003). Examining the order in which items are recalled can reveal memory organization, reflecting how individuals search for and extract events in their memory systems. It has been found that memory extraction processes can be categorized into temporal clustering (temporally closer items are more likely to be recalled successively (Kahana, 1996)), spatial clustering (geographically closer items are more likely to be recalled successively (Miller et al., 2013a, 2013b; Pathman et al., 2023)), and semantic clustering (items are more likely to be recalled successively (Kahana, 2020; Manning & Kahana, 2012)). Based on the findings of temporal clustering, researchers have termed the phenomenon that temporally close events are more likely to be linked together during memory and learning, thus affecting memory retention and recall, the temporal contiguity effect (TCE) (Sederberg et al., 2010). The temporal contiguity effect is so robust that researchers have referred to it as a fundamental property of the memory system (Healey et al., 2019; Healey & Kahana, 2014; Kelly & Beran, 2021), and the magnitude of the temporal contiguity effect predicts subjects’ recall performance, i.e., the larger the temporal contiguity effect, the more items that are recalled (Sederberg et al., 2010; Healey et al., 2019; Healey & Kahana, 2014; Spillers & Unsworth, 2011). Previous studies have examined individuals’ temporal sequential memory (Kahana, 1996; Miller et al., 2013b; Spillers & Unsworth, 2011; Ward et al., 2010). Kelley et al. (2014), examining the effects of collaborative memory and partial cues on collaborative memory, first examined temporal sequential memory for collaboration and found that collaboration inhibits temporal sequential memory.

Most real-world memory activities are inherently social in nature (Kelly & Beran, 2021), yet the group differences induced by JOL cannot be adequately explained by traditional individual-level memory theories. This gap motivates the examination of collaborative memory—the process where individuals work together in a group to remember information, which differs from individual memory in several key aspects. Specifically, in collaborative memory paradigms, participants typically study material individually before recalling it collectively as a group (Kelly & Beran, 2021). Collaboration can be beneficial through post-collaborative facilitation and error correction, but it may also lead to collaborative inhibition (Rajaram & Maswood, 2017). Weldon and Bellinger (1997) first found that the total amount of information extracted by group members together is more than any one individual alone but less than the total amount of information extracted by an equal number of individuals alone, a phenomenon he called collaborative inhibition (Rajaram & Maswood, 2017). The mainstream explanation for collaborative inhibition is the retrieval strategy disruption hypothesis (Basden et al., 1997). Basden et al. (1997) proposed this hypothesis. He argued that individuals have unique knowledge experiences and organizational structures formed during learning. For example, when learning the words “apple”, “plum”, “banana”, and “durian”, Person A’s memory organization might be “apple, banana, durian, plum”. In contrast, Person B’s organization could be “apple, durian, plum, banana”. When A and B collaborate to recall these words, their different organizational structures interfere, affecting their memory performance. Nominal groups (consisting of an equal number of members with the collaborative group, nominally a group, but actually extracting individually) avoid this interference. Therefore, collaborative groups’ retrieval performance is lower than nominal groups, leading to collaborative inhibition (Basden et al., 1997). It has been demonstrated that collaborative inhibition exists whether in free recall (Barber et al., 2010; Basden et al., 1997, 2000; Rajaram & Maswood, 2017), recognition (Abel & Bäuml, 2020, 2023), or sequential memory (Kelley et al., 2014). Collaborative inhibition is a consistent phenomenon observed in collaborative memory. However, previous studies have primarily focused on standard learning of materials, and it remains unclear whether the addition of judgments of learning to the learning process would influence collaborative inhibition. Suppose JOLs can produce positive reactivity effects. In that case, the enhancement effect of JOLs on collaborative groups may potentially exceed that on nominal groups, thereby offsetting the negative impact (i.e., collaborative inhibition) inherent in collaborative settings. However, JOL not only affects the content of memory (i.e., items) but also impacts the organization of memory (i.e., inter-item relationships).

Zhao et al. (2023) discovered that in individual memory, the judgment of learning (JOL) has a dissociative reactivity effect on item and inter-item relational memory. They discovered that making judgments of learning (JOLs) can promote item-specific memory but simultaneously disrupt the memory of temporal relationships between items. In this study, participants were required to learn multiple lists of Chinese words (each list containing 12 words). During the learning phase, half of the lists were presented under the JOL condition, while the other half were under the no-JOL condition. Under the JOL condition, a slider ranging from 0 to 100 was presented below each learning word. Participants were required to predict the likelihood of successfully remembering the word in a subsequent test while learning it. Under the no-JOL condition, participants only needed to learn each word without making JOLs. After learning all the word lists, participants were required to complete both a recognition test and an order reconstruction test. The results showed that compared with the no-JOL condition, making JOLs during learning significantly improved participants’ recognition memory performance, indicating that JOLs promoted item-specific memory and had a positive effect. However, participants in the JOL condition had a worse recall of the learning order of different words, indicating that JOL disrupted the memory of temporal relationships between items and had a negative effect. Specifically, JOL enhances memory for individual items but disrupts the ability to recall those items’ temporal order, highlighting the dissociative reactivity effects. The theoretical foundation for these dissociative reactivity effects is grounded in two hypotheses: the relational and item-specific processing hypothesis and the item-order account.

The relational and item-specific processing hypotheses suggest that memory can be enhanced by two forms of information processing: relational and item-specific processing. Relational processing involves encoding similarities or relationships between events. In contrast, individual item-specific processing involves encoding information unique to each event (Hunt & Einstein, 1981). Researchers believe that JOL induces processing specific to the item of memorized information because when learners make JOLs, they focus on the current specific item, thus enhancing the uniqueness of each item (Hunt & Einstein, 1981; Hunt & Seta, 1984). In other words, making judgments of learning is akin to engaging in an information encoding task, and adding such tasks tends to enhance item-specific processing (Hunt, 2013; Hunt & Einstein, 1981; Hunt & Seta, 1984). This hypothesis is also supported by several studies (Mitchum et al., 2016; Schmidt & Schmidt, 2016). The item-order hypothesis suggests that the performance of free recall is influenced not only by the characteristics of individual items but also by inter-item relations, especially by serial order information. It also states that item memory and memory for inter-item relations are separable (McDaniel & Bugg, 2008). When a given encoding strategy enhances the processing of item-specific information, inter-item relational processing may be disrupted because more cognitive resources are allocated to item-specific processing. In contrast, inter-item relational processing is reserved for fewer cognitive resources. Zhao et al. (2023) confirmed the dissociable reactivity effect of judgments of learning on items and inter-item relational memory. However, the study’s subject group comprised students from the most prestigious schools. It remains to be verified whether this phenomenon is also present among students from other educational levels. Additionally, Zhao et al. (2023) focused on individual memory, so further investigation is needed to determine if the dissociable reactivity effect also exists in collaborative memory. As we know, collaborative inhibition is a robust phenomenon where collaborative retrieval not only results in poorer item memory performance compared with nominal groups but also leads to inferior memory organization, as measured by the adjusted ratio of clustering (ARC). If judgments of learning (JOLs) disrupt memory organization, both collaborative and nominal groups should exhibit impaired performance. Under such conditions, the detrimental effect of collaborative retrieval on memory organization may become less pronounced. This could eliminate the performance disparity between the two groups and cause the collaborative inhibition effect to disappear. The present study will employ empirical methods to investigate whether collaborative inhibition vanishes under the JOL condition.

This study employed a 2 (group: collaborative vs. nominal; between-subjects) × 2 (metamemory monitoring: with vs. without judgments of learning; within-subjects) × 2 (test type: recognition vs. sequential reconstruction; within-subjects) mixed factorial design. The dependent variables assessed were recognition scores and sequential reconstruction scores. In a recognition test, we choose a forced-choice recognition task, where learned words and unlearned words appear in pairs. The task for participants is to determine which word has been learned. The forced-choice recognition task is primarily used to examine whether JOL can enhance item-specific processing. According to the item-order account, JOL will enhance item-specific processing (McDaniel & Bugg, 2008). However, the dual-task costs (encoding plus making JOLs) suggest that making JOLs might borrow limited cognitive resources from the primary learning task, leading to a weaker encoding of temporal information (Mitchum et al., 2016). Accordingly, the item-order account predicts a positive reactivity effect on recognition. By contrast, the dual-task costs explanation predicts a negative reactivity effect on recognition. If the results show that the recognition accuracy rate for JOL is higher than that for no-JOL, it supports the item-order account. Conversely, if the recognition accuracy rate for JOL is lower than that for no-JOL, it indicates support for the dual-task costs explanation. Through the accuracy rate as an indicator, we can illustrate the impact of JOLs on item processing. Therefore, in this study, we only analyzed the recognition accuracy rate and did not conduct analyses on false alarm rates and d’.

The dissociative effect of JOL on item vs. sequential memory has only been tested in individual contexts (Zhao et al., 2023), leaving its manifestation in collaborative settings unexplored. No study has examined whether JOL’s positive item-memory effects could mitigate collaborative inhibition—a robust phenomenon where group recall underperforms nominal groups (Basden et al., 1997; Weldon & Bellinger, 1997). The study investigated the reactivity effects of JOLs on both the items and the order of collaborative memory to test the item-order hypothesis. Specifically, it first aimed to assess whether JOL has a reactivity effect on word list learning in collaborative memory, determine the reactivity effect of JOL on temporal-order memory during collaborative recall, and finally compare the differences between collaborative and non-collaborative memory in terms of item and temporal-order memory when JOL is applied. Existing research suggests that JOLs can have a positive reactivity effect on item memory (Dougherty et al., 2018; Mitchum et al., 2016; Zhao et al., 2022).

**Hypothesis** **1.** *The correct recognition rate in collaborative and nominal groups was higher in the JOL condition than in the no-JOL condition. According to previous research, JOL produces a negative reactivity effect on sequential memory (Zhao et al., 2023).*

**Hypothesis** **2.** *Sequential reconstruction correctness in collaborative and nominal groups was lower in the JOL condition than in the no-JOL condition.*

## 2. Methodology

### 2.1. Data Collection and Sample

Referring to the previous study (Abel & Bäuml, 2020), G-Power 3.1 software was utilized to determine the sample size for a repeated measures ANOVA test. The parameters were set to achieve a statistical power of 0.8 at a significance level of 0.05, with a medium effect size *f* = 0.25 and a correlation among repeated measures of 0.50. The calculation indicated that 34 subjects were required. Because the experiment was conducted in groups of two, a total of 68 subjects were needed. Seventy-six college students from a higher education institution were randomly selected and paired into two groups. They were then randomly assigned to either collaborative or nominal groups. There were 19 groups in the nominal group (38 participants) and 19 in the collaborative group (38 participants). However, two groups from the collaborative group were excluded because their scores exceeded three standard deviations. Consequently, the final number of valid subjects was 72 (28 females).

The paradigm of collaborative memory involves using collaboration groups and nominal groups for comparison. To investigate whether collaborative cooperation between two individuals yields better memory effects compared with individuals working alone, a control group was established. The control group was merely called a group in name only; in reality, the two individuals were learning independently of each other. The purpose is to examine which method, collaborative learning between two people or individual learning, is more effective. This study employed a completely randomized method for group division.

The nominal group consisted of 19 groups, with an average age of 19.58 years (*SD* = 0.86), while the collaborative group had 17 groups, also with an average age of 19.58 years (*SD* = 0.86). There was no significant difference in age between the two groups, as indicated by *t*_(70)_ = 0.89, *p* = 0.38, *Cohen’s d* = 0.21. Subjects had normal naked or corrected visual acuity and no color blindness or color deficiency. All participants were unaware of the true purpose of the experiment. All participants provided written informed consent to participate in this study, which the Ethics Review Committee of Tianjin Normal University approved. Participants received appropriate monetary compensation for their participation. Recruitment start date: 15 June 2024; recruitment end date: 15 July 2024.

### 2.2. Experimental Materials

Irrelevant word lists were employed as the experimental material in this study because temporal-order information plays a crucial role in recall for semantically irrelevant list learning tasks (Spillers & Unsworth, 2011; Unsworth, 2008). When memorizing materials, our memory organization methods include semantic clustering, temporal clustering, and spatial clustering, among others. Semantic clustering refers to the phenomenon where, during the recall process, people tend to recall semantically related items consecutively. Temporal clustering refers to the phenomenon where, during the recall process, people tend to recall adjacent items that were learned together. When we select irrelevant word pairs, we avoid individual semantic clustering. Then, during the learning process, the subjects are more likely to adopt temporal clustering.

Drawing from previous research (Zhao et al., 2023), 226 double-character words with word frequencies ranging from 2.98 to 35.62/million were selected from the Chinese lexicon developed by Cai and Brysbaert (2010). Prior to the formal experiment, 28 college students (who did not participate in the formal experiment) were asked to rate the familiarity and difficulty of 226 words on a seven-point scale, and 152 words with familiarity ranging from 4.59 to 6.19 and difficulty ranging from 2.57 to 3.16 were selected as the experimental materials. Eight words were used for practice. In contrast, the remaining 96 words were used for the formal experiment, and 48 words were used as the new words for the recognition test. An utterly randomized method was employed to control for extraneous variables. For each group of subjects, 144 words were randomly divided into two sets. A total of 96 words were designated as “old”, and 48 words were categorized as “new”. The 96 old words were split into eight lists, with four lists designated for learning under the judgment of learning (JOL) condition and four reserved for the no-JOL condition. The order of presentation for the eight lists was randomized, the order of the JOL was randomized, and the order of the 12 words within each list was also randomized. For computational purposes, two participants within a nominal group learned the same materials in the same order. In comparison, any two participants in different nominal groups learned the materials in a manner that was distinct from one another.

### 2.3. Experimental Design

A 2 (group: collaborative vs. nominal; between-subjects) × 2 (metamemory monitoring: with vs. without judgments of learning; within-subjects) × 2 (test type: recognition vs. sequential reconstruction; within-subjects) mixed factorial design was employed. The dependent variables measured were recognition correctness and sequential reconstruction correctness.

### 2.4. Experimental Procedure

The experimental procedure follows the experimental paradigm of Zhao et al. (2023). Subjects completed the tests individually or in pairs in a separate room. The experimental apparatus was two 14-inch laptops with a screen resolution of 1024 × 768. The experimental program was written and presented using E-prime 3.0. Subjects were informed before the experiment that they needed to perform a memory task. The collaborative group was informed that they had a partner. They studied individually and then worked together to complete the recognition test and the sequential reconstruction task. The nominal group was not informed about a partner; they studied alone and independently completed the recognition test and sequential reconstruction. Participants first underwent a complete round of practice with the experimental procedure, practicing four words in the JOL condition and four in the no-JOL condition. The formal experiment was conducted only after the participants fully understood the experimental procedure.

The formal experimental procedure is as follows:

Learning task: Subjects were required to learn eight lists with a total of 96 words. Four lists required JOL (JOL condition), and four lists did not require JOL (no-JOL condition), and prior to the presentation of each list, subjects were informed whether this list required JOL or not. In the word list that did not require judgments of learning (JOLs), a 500 ms gaze point was first presented, followed by the sequential presentation of the 12 words in the list. Each word was presented for 5000 ms, with a 500 ms gaze point as the interstimulus interval between words. In the list of words that required JOL, the gaze point was presented first (500 ms), followed by the word (5000 ms), and a slider bar appeared below each word, asking subjects to predict how likely they would be able to recall the word on a subsequent memory test on a scale of 0 (surely cannot remember it)–100 (definitely remember) rating; the higher the score means, the higher the likelihood of being able to recall the word. The mouse clicks on the corresponding number of the slider to complete the memory prediction, and the screen will present a 5 s countdown; the subject needs to complete the prediction within 5 s in order to ensure that the JOL condition and the no-JOL condition present the same amount of time. Even if the subject completes the prediction within 5 s, the word on the screen will continue to be presented until the end of the 5 s. The next word was presented, and each word was accompanied by a 500 ms gaze point before presentation as the stimulus interval.

Distraction task: After the learning task, subjects were required to complete a 3 min distraction task in which a three-digit number was randomly presented on the screen. Subjects were required to perform consecutive minus three-digit calculations and write down the answer to each calculation step on an A4 sheet of paper.

Recognition test: After the distraction task, subjects needed to complete two memory tests: a forced-choice recognition task and sequential reconstruction. The order of the two tests was randomized. Four learned lists (2 JOL conditional lists and 2 no-JOL conditional lists) were randomly selected for the recognition test, and the other four lists were tested for sequential reconstruction. During forced-choice recognition task test, old words (48) and new words (48) were presented in random pairs, and subjects were required to decide which of the two words was the old learned word, with no time limit.

Sequential reconstruction test: Four lists were presented randomly. Twelve words from each list were simultaneously presented on the screen in a random order. The subjects needed to recall the original presentation order of these 12 words and fill in the words in the positions corresponding to the serial numbers 1–12 on the A4 paper, with unlimited time. To avoid the order effect, half of the participants first took the recognition test, while the other half took the sequence reconstruction test first.

**Figure 1:**
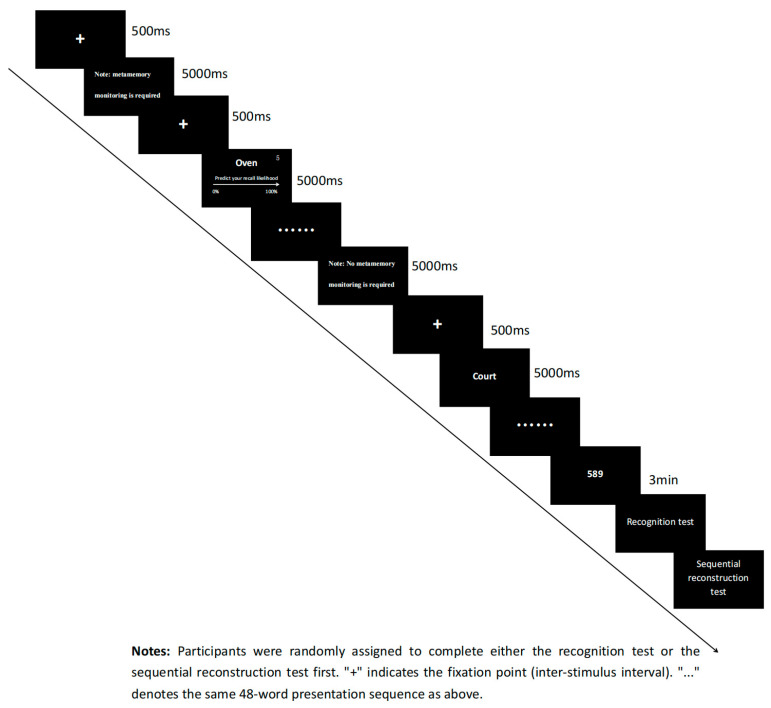
Experimental flowchart.

The collaborative group consisted of pairs of individuals who discussed together to complete the recognition and order reconstruction test. There were no restrictions on the time or manner of their discussion. In contrast, the nominal group participants completed the process individually, with no time restrictions (Figure 1).

### 2.5. The Method of Scoring Recognition and Reconstruction Tasks

For the collaborative group, consisting of two individuals, the score was calculated as follows: After discussion, they provided a joint answer. The total number of correct recognitions or correct sequences created by the two individuals, divided by the total number of learning items, represents the recognition score and the sequence reconstruction score, respectively. For the nominal group, the recognition score was calculated by summing the number of items correctly recognized by Group Member A and those correctly recognized by Group Member B, then subtracting the number of items correctly recognized by both members and dividing by the total number of items studied by the two participants. For instance, if both participants studied the same set of four words—“apple, banana, peach, plum”—and Member A correctly recognized “apple” and “banana” while Member B correctly recognized “banana” and “peach”, the recognition score would be computed as follows: the sum of A’s correct recognition (2 items: “apple” and “banana”) and B’s correct recognition (2 items: “banana” and “peach”), minus the number of items correctly recognized by both A and B (1 item: “banana”), divided by the total number of studied items (4 items: “apple, banana, peach, plum”). This calculation yields a recognition score of 75%. The order reconstruction score was calculated similarly: the sum of the number of items correctly ordered by each participant minus the number of items correctly ordered by both participants, divided by the total number of items.

## 3. Results

A comparison was conducted between the collaborative and nominal groups to identify differences in their recognition and sequential reconstruction processes under JOL conditions. The retrieval accuracy rates under different conditions are presented in Table 1.

A three-way repeated measures ANOVA test was conducted on a 2 (group: collaborative vs. nominal) × 2 (metamemory monitoring: with vs. without judgments of learning) × 2 (test type: recognition vs. sequential reconstruction) design with recall correctness as the dependent variable. 

The results showed there was a significant main effect of group (F_(1,34)_ = 9.78, *p* < 0.01, *η*^2^ = 0.22), with the nominal group scoring greater than the collaborative group. There was a significant main effect of the JOL condition (F_(1,34)_ = 8.68, *p* < 0.01, *η*^2^ = 0.20), with the JOL condition scoring more significant than the no-JOL condition in terms of percentage correct. There was a significant main effect of test type (F_(1,34)_ = 1463.20, *p* < 0.01, *η*^2^ = 0.98), and recognition scores were higher than sequential reconstruction scores.

The third-order interaction was nonsignificant (F_(1,34)_ = 0.01, *p* = 0.91, *η*^2^ = 0.001). For the two-way interactions: two-order interaction results: group × JOL condition: nonsignificant (F_(1,34)_ = 0.67, *p* = 0.42, *η*^2^ = 0.02), group × test type: nonsignificant (F_(1,34)_ = 0.27, *p* = 0.61, *η*^2^ = 0.01), and JOL condition × test type: significant (F_(1,34)_ = 11.39, *p* < 0.01, *η*^2^ = 0.25).

**Figure 2:**
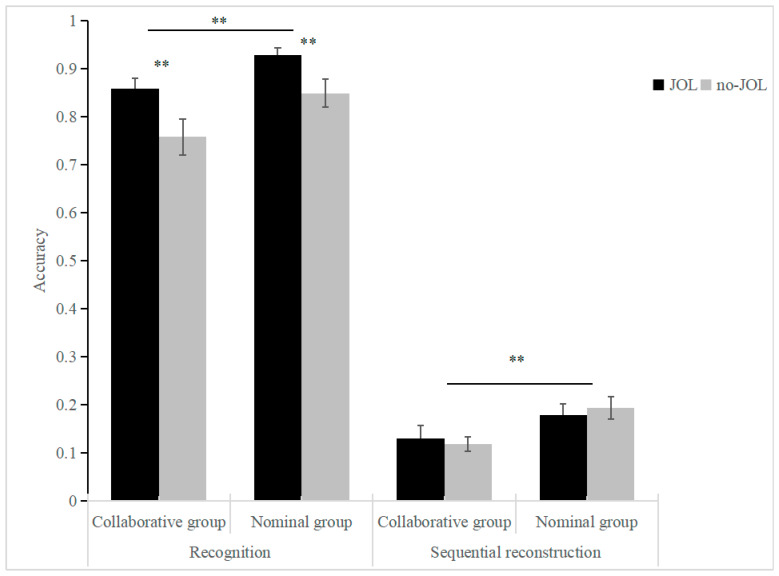
The accuracy of recognition and sequential reconstruction in different experimental conditions (** *p* < 0.01).

To align with the research objectives, planned comparisons were conducted using paired *t*-tests to compare accuracy rates under different test types for the two types of metamemory monitoring. Under the recognition condition, the difference between the JOL and no-JOL conditions was significant (*t*_(35)_ = 4.24, *p* < 0.001, *cohen’s d* = 0.71). Recognition performance was higher in the JOL condition than in the no-JOL condition (*difference* = 0.09, *95%CI* = [0.05 0.13]). Under the sequential reconstruction condition, the difference between the JOL and no-JOL conditions was not significant (*t*_(35)_ = −0.12, *p* = 0.91, *cohen’s d* = −0.02). This result suggests that sequential reconstruction performance was unaffected by JOL. (See Figure 2).

## 4. Discussion

This study investigated how judgments of learning (JOLs) affect memory performance across different task types (recognition vs. sequential reconstruction memory) within collaborative memory contexts. Our results demonstrated a significant reactivity effect of JOLs on recognition tasks, consistent with previous findings (Rivers et al., 2023; Spellman & Bjork, 1992). Soderstrom et al. (2015) proposed a cue-strengthening theory to explain the reactivity effect in paired-associate learning. Soderstrom and colleagues observed that making JOLs significantly enhanced memory for related word pairs, but the reactivity effect on paired-associate learning was minimal. However, in this study, irrelevant words were used as stimuli, and the positive reactivity effect of JOL was still observed, which cannot be explained by the cue-strengthening theory. The goal-changed hypothesis suggests that individuals adjust their learning goals when performing JOL to avoid futile attempts to memorize difficult items. They shift from trying to learn all items to focusing on easier ones at the expense of more challenging ones. This strategy results in more efficient memorization and improved memory performance (Mitchum et al., 2016). However, the learning materials used in the current study were irrelevant word lists and controlled for difficulty. What factors cause subjects to perceive differences in difficulty despite word lists of the same difficulty level, leading them to learn more of the words they perceive as easy at the expense of those they perceive as brutal, resulting in a positive reactivity effect? Was it that participants still autonomously selected which words to remember first, choosing those they perceived as simpler, even though the difficulty of the words was controlled? Or is it possible that the goal-changed hypothesis may not be the best explanation for the current study? This remains uncertain at present. Another classic theory, the item-specificity hypothesis, can explain this phenomenon. The item-specificity hypothesis suggests that when learners are making JOLs, they are prompted to process the uniqueness of the information they memorize deeply. While making JOLs, learners focus their attention on these specific items, which enhances the uniqueness of each item, and memory performance increases, demonstrating the reactivity effect of JOL. This study found that JOL promotes collaborative memory. This study extends JOL’s reactivity effect to the collaborative memory field. It verifies that the reactivity effect of JOL also exists in collaborative memory, which is one of the contributions of this study.

This study found that JOL did not affect sequential reconstruction performance. There was no difference in sequential memory performance between the JOL group and the no-JOL group, which is inconsistent with the view of the item-order hypothesis (McDaniel & Bugg, 2008): according to the item-order hypothesis, item memory is better in the JOL group. Sequential memory should be worse, but the results did not confirm this hypothesis. Possible reasons for this are that sequential memorization is too tricky, overall correctness is lower, and a floor effect occurs. The mean correct rate of individual memory in this study was 0.1 in both the JOL and no-JOL groups, while in the previous study, the mean correct rate was 0.15 in the JOL group and 0.21 in the no-JOL group (Zhao et al., 2023). The experimental procedures of the two studies were identical, and the main reason for the difference in the correct rate may be the subject difference. The subjects in this study were college students from vocational colleges. In contrast, the subjects in the Zhao et al. (2023) study were college students from major universities, so there may be a difference in memorization ability. Of course, it is also possible that JOL produces a separate response to items and sequences, which requires a boundary condition, and that no effect on sequential memory occurs when the difficulty is too high. However, this boundary condition needs to be verified by further experimental studies. On the other hand, although no adverse reactivity effects were found in sequential memory, no positive effects were found either, suggesting that items and sequences are indeed separated. According to item-specificity theory, making judgments of learning enhances the depth of processing of current item uniqueness, which did not show positive reactivity effects in sequential reconstruction, suggesting that the effects of judgments of learning on sequential and item memory are separate.

The dissociative effects of JOLs on item memory and sequential memory have significant educational and practical implications. In terms of teaching strategies, one can adopt personalized learning, where teachers can provide personalized learning suggestions and resources based on students’ performance in JOLs. For students who struggle with sequential memory, specific exercises can be designed to enhance their ability to remember sequences. Regarding diversified teaching methods, teachers can combine various teaching methods, such as independent study, group discussion, and interactive learning, to meet the needs of different students. These methods can better leverage the advantages of JOL while mitigating its negative impact on sequential memory. When designing learning materials, teachers can clearly distinguish between individual items that need to be remembered and the sequential relationships that require memorization. For individual items, a JOL component can be added; for sequential relationships, specific exercises can be designed to strengthen memory. Through JOL, teachers can promptly understand students’ learning situations and provide targeted feedback and tutoring. This feedback mechanism can help students adjust their learning strategies promptly, thereby improving their learning outcomes. During the exam preparation stage, students can use JOL to assess their mastery of each knowledge point, thereby making reviewing more targeted. For content that requires remembering sequential relationships, specific exercises can be designed to strengthen memory and recall. Online learning platforms can incorporate JOL functionality to enable students to conduct self-assessments during the learning process. Platforms can provide personalized learning suggestions and resources based on students’ JOL results, thereby enhancing learning outcomes.

Meanwhile, the present study also found that in collaborative memory, the collaborative group and the nominal group did not show differences in sequential memory under both JOL and no-JOL, suggesting that individual and collaborative memory, in the domain of sequential memory, may have similar mechanisms. The presence of collaborative inhibition was found in both the recognition and sequential reconstruction tests, i.e., the nominal group’s recognition scores were higher than those of the collaborative group, which is consistent with previous findings (Abel & Bäuml, 2023; Kelley et al., 2014), and this result also suggests that collaborative inhibition is indeed a stable memory phenomenon. This result can be explained by the retrieval strategy disruption hypothesis (Basden et al., 1997), in which subjects each have their unique way of processing information in collaborative memory, and during collaborative retrieval, the subjects’ unique memory strategies are disrupted, which affects the memory performance, suggesting that collaborative memory not only interferes with an individual’s memory for items but also interferes with sequential memory.

The present study found no interaction between grouping types and metamemory judgments, suggesting that reactivity effects and collaborative inhibition are two independent memory phenomena. This independence may stem from their distinct mechanisms: JOL (judgment of learning) primarily operates during encoding by enhancing item-specific processing (Senkova & Otani, 2021), whereas collaborative memory affects individuals at retrieval (Basden et al., 1997). Specifically, collaborative retrieval disrupts individuals’ ability to employ optimal retrieval strategies, leading to poorer performance in collaborative groups compared with nominal groups. These divergent mechanisms explain the independence between reactivity effects and collaborative inhibition.

This study examined JOL’s effects on recognition and sequential reconstruction in collaborative memory. Theoretically, it pioneers the investigation of JOL’s dual effects in collaborative memory research, enriching the field’s theoretical framework. Practically, the findings offer actionable insights for educators: For item memory (e.g., vocabulary, facts), integrating JOL during learning enhances retention by promoting item-specific processing. For sequential memory (e.g., timelines, procedures), instructors should prioritize sequential techniques (e.g., chunking, rehearsal) to compensate for JOL’s limitations. Such tailored strategies can mitigate collaborative retrieval’s negative impacts on performance.

This study investigated the effects of JOL on recognition and sequential reconstruction memory in collaborative memory, which holds profound significance. From a theoretical perspective, this study is the first to introduce the impact of metacognitive monitoring through JOL on recognition and sequential reconstruction memory in the field of collaborative memory, thereby further enriching the theoretical framework of collaborative memory. From a practical standpoint, exploring the effects of JOL on recognition and sequential reconstruction memory in collaborative memory can better guide teachers’ instruction. This can be specifically implemented in the following ways: Firstly, for enhancing students’ self-assessment abilities by guiding them to conduct JOL during the learning process to evaluate their mastery of learning materials, which can facilitate the memory of items. Secondly, for optimizing the organization of group learning by reducing collaborative inhibition and leveraging the advantages of JOL, for example, by having students complete learning tasks independently before sharing and discussing within the group. And thirdly, for strengthening sequential memory instruction by designing specific exercises to enhance students’ sequential memory abilities, such as creating fill-in-the-blank or sequencing questions when teaching content that requires sequential memorization to reinforce the learning of sequences. The limitations of this study are as follows: Firstly, the small number of participants may result in insufficient data. Secondly, the low sequential reconstruction scores may indicate a floor effect. Thirdly, the participants were limited to a small number of vocational college students, which lacks sufficient demographic diversity and thus limits the generalizability of the findings. Building on this study, future research could investigate whether JOL judgments also exhibit dissociation between item memory and semantic relational memory. Additionally, JOL could be further applied to research on false memories. A typical paradigm for false memory research involves participants first learning a series of semantically related words (such as “bed,” “rest,” “dream,” and “doze”), which are all associated with a critical lure word (such as “sleep”) that is not present in the learning list. In the subsequent recall test, participants often falsely recall the critical lure word, thereby generating a false memory. If JOL judgments can weaken the memory of relationships between items, then JOL judgments may reduce the likelihood of false memories.

## 5. Conclusions

This study demonstrates that collaborative inhibition occurs in collaborative memory during recognition and sequential reconstruction. No reactivity effect of JOL was observed in the sequential reconstruction test. Additionally, the positive reactivity effect of JOL item on recognition memory did not differ between the nominal and collaborative groups, suggesting that the reactivity effect of JOL and the collaborative inhibition effect are independent and do not influence one another. The dual-process model of memory may explain this discovery. The dual-process model posits that the memory process consists of two distinct mechanisms: automatic processing (such as the extraction of perceptual features) and controlled processing (such as strategic encoding and retrieval) (Evans & Stanovich, 2013). The independence of JOL’s reactive effect and the collaborative inhibition effect indicates that these two effects correspond to different processing mechanisms. Specifically, JOL’s reactive effect may be more similar to controlled processing, as it involves individuals adjusting their learning and retrieval strategies for memory items. In contrast, the collaborative inhibition effect may be related to automatic processing, as it involves unconscious processing and interference of information in a group environment. The mutual independence of JOL’s reactive effect and the collaborative inhibition effect in this study provides empirical evidence for the different processing mechanisms in the memory process.

## Figures and Tables

**Table 1:** Retrieval accuracy under various conditions.

Condition	*N* (Number of Groups)	Recognition Correctness (*M* ± *SD*)		Sequential Reconstruction Correctness (*M* ± *SD*)	
		JOL	No-JOL	JOL	No-JOL
Collaborative group	17	0.86 ± 0.09	0.76 ± 0.16	0.13 ± 0.11	0.12 ± 0.06
Nominal group	19	0.93 ± 0.07	0.85 ± 0.15	0.18 ± 0.10	0.19 ± 0.10

## Data Availability

The research data are available in Supplementary Materials.

## Supplementary Materials

The following supporting information can be downloaded at: https://www.mdpi.com/article/10.3390/bs15070905/s1.
